# Variability of Pyrrolizidine Alkaloid Occurrence in Species of the Grass Subfamily Pooideae (Poaceae)

**DOI:** 10.3389/fpls.2017.02046

**Published:** 2017-11-30

**Authors:** Anne-Maria Wesseling, Tobias J. Demetrowitsch, Karin Schwarz, Dietrich Ober

**Affiliations:** ^1^Biochemical Ecology and Molecular Evolution, Botanical Institute and Botanic Gardens, University of Kiel, Kiel, Germany; ^2^Department of Food Technology, Institute of Human Nutrition and Food Science, University of Kiel, Kiel, Germany

**Keywords:** Pooideae, LC-QTOF-MS, SCX-SPE, pyrrolizidine alkaloid, thesinine, thesinine-glycoside

## Abstract

Pyrrolizidine alkaloids (PAs) are a class of secondary metabolites found in various unrelated angiosperm lineages including cool-season grasses (Poaceae, subfamily Pooideae). Thesinine conjugates, saturated forms of PA that are regarded as non-toxic, have been described to occur in the two grass species *Lolium perenne* and *Festuca arundinacea* (Poaceae, subfamily Pooideae). In a wider screen, we tested various species of the Pooideae lineage, grown under controlled conditions, for their ability to produce thesinine conjugates or related structures. Using an LC-MS based targeted metabolomics approach we were able to show that PA biosynthesis in grasses is limited to a group of very closely related Pooideae species that produce a limited diversity of PA structures. High variability in PA levels was observed even between individuals of the same species. These individual accumulation patterns are discussed with respect to a possible function and evolution of this type of alkaloid.

## Introduction

Pyrrolizidine alkaloids (PAs) are a typical class of plant secondary (or specialized) metabolites produced in various lineages of the flowering plants and their occurrence, structural diversity, and biosynthesis have been studied extensively for several decades (Hartmann and Witte, 1995; Stegelmeier et al., 1999; Ober and Kaltenegger, 2009). In cool-season grasses (Poaceae, subfamily Pooideae) a specific class of PAs, known as lolines, have been described to accumulate in individuals infected with specific endophytic fungal symbionts that were shown to synthesize these alkaloids (for review see Schardl et al., 2007).

Koulman et al. (2008) analyzed grass extracts and detected PAs that are the product of the plant’s own metabolism. The authors were able to show that, in the pasture grass species *Lolium perenne* and *Festuca arundinacea*, specific PA conjugates are detectable irrespective of fungal infection (Koulman et al., 2008). Structure elucidation revealed these compounds to be the stereoisomers *E*- and *Z*-thesinine in varying states of glycosylation. In addition to the aglycone, the rhamnoside and a glycosylation product of the rhamnoside named rhamnoside-glycoside were described (Koulman et al., 2008). PAs from several dicotyledonous species are, due to their toxicity, regarded as defensive compounds protecting the plant from herbivores (Ober, 2003; Fu et al., 2004; Langel and Ober, 2011). However, 1,2-saturated PAs such as thesinine found in the grasses are considered to be non-toxic (Hartmann and Witte, 1995). For this reason and the fact that these alkaloids accumulate in cultivars bred as pasture grasses to feed livestock, it was concluded that these compounds are not part of the plant’s chemical defense (Koulman et al., 2008). Therefore, the biological function of these glycosylated grass PAs remains enigmatic.

Pyrrolizidine alkaloid biosynthesis relies on the presence of a homospermidine synthase (HSS), which catalyzes the first pathway-specific step and, thereby, connects primary with secondary metabolism (Ober et al., 2003). HSS has its origin in the duplication of a gene encoding deoxyhypusine synthase (DHS), an enzyme of primary metabolism involved in the post-translational activation of the eukaryotic initiation factor 5A (Park, 2006; Park et al., 2010). In a recent study *hss* genes have been identified from *L. perenne* and *F. arundinacea* that originated from a gene duplication event that took place during Pooideae species radiation. Of note, several other extant Pooideae species have retained functional *hss* genes in their genomes. In addition to the aforementioned pasture grasses, the Pooideae also include important crop species such as bread wheat (*Triticum aestivum*), for which also a functional HSS was detected (Wesseling et al., unpublished data). As the expression of HSS in members of the Pooideae lineage suggests a general capacity to produce PAs, we decided to screen, in addition to *L. perenne* and *F. arundinacea*, further members of this taxonomic group for the presence of PAs.

We have grown various grass species under controlled conditions and tested them for the presence of PAs by using an LC-MS-based targeted metabolomics approach. Knowledge about the occurrence of PAs and analysis of variation in alkaloid levels in this plant lineage should allow a better general understanding of the phytochemistry and ecology of PA biosynthesis in grasses.

## Results

### Approach for the Detection of Thesinine and Its Conjugates via LC-QTOF-MS

**FIGURE 1:**
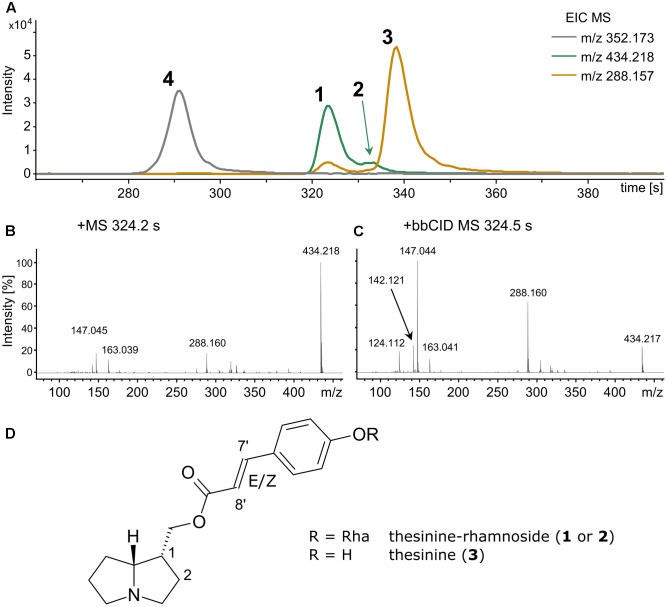
Thesinine and thesinine-rhamnosides detected in a *Festuca arundinacea* extract via LC- QToF-MS analysis. Extracted ion chromatograms (EICs, smoothed, **A**) depicting the elution profiles of thesinine-rhamnosides (434.218 m/z, **1** and **2**), thesinine (288.157 m/z, **3**), and the internal standard retrorsine (352.173 m/z, **4**). Mass (MS, **B**) and broadband fragmentation spectrum (MSn, **C**) recorded at retention time 323 s (asterisk). **(D)** Structure of E/Z-thesinine-conjugate.

To detect thesinine and its conjugates crude grass extracts were analyzed by LC-QTOF-MS. The compounds could readily be identified by their respective [M+H]^+^ ions, namely *m/z* 288.159 for thesinine (M: C_17_H_21_NO_3_) and *m/z* 434.217 for thesinine-rhamnoside (M: C_23_H_31_NO_7_) (**Figure 1**). Broadband MSn fragmentation provided further diagnostic ions, namely *m/z* 142.121 and *m/z* 124.112, representing the necine base of thesinine and one of its typical fragments, respectively (**Figure 1**). In accordance with the findings of Koulman et al. (2008), who identified *E*- and *Z*-stereoisomers of thesinine-rhamnoside, we observed two distinct *m/z* 434.2 peaks in the chromatogram with retention times (t_R_) of 324 s and 334 s (**Figure 1A** and Supplementary Figure S1). In our LC-MS setup the aglycone thesinine was shown to elute after the thesinine-rhamnosides at 340 s (**Figure 1** and Supplementary Figure S1). Stereoisomers for a disaccharide conjugate of thesinine (*m/z* 549) were only present in traces in our LC-MS analyses (maximal 1000 counts) and only in samples that also contained the monosaccharide thesinine-rhamnosides (up to about 70,000 counts). Therefore, detailed analyses of the disaccharide conjugates would not provide any additional information on the presence or absence of PAs in grasses and were not further addressed in this study. Furthermore, PAs are known to occur as either tertiary amines or *N*-oxides (Hartmann and Witte, 1995; Stegelmeier et al., 1999). The thesinine-conjugates identified by Koulman et al. (2008) are tertiary amines. Testing for the respective *N*-oxides (calculated *m/z*: 304.155 and 450.213 for the hypothetical thesinine-*N*-oxide and thesinine-*N*-oxide-rhamnoside, respectively) revealed that these were not present in the extracts.

### Occurrence of Thesinine and Its Conjugates Is Limited to Specific Pooideae Species

Initial LC-MS analyses of extracts obtained from *T. aestivum*, *Dactylis glomerata*, and *Holcus mollis* (all Pooideae, Poaceae) suggested the absence of thesinine-conjugates in these species (results not shown). Therefore, we decided to focus on the *Lolium*-*Festuca* species complex as defined in the literature for further tests on PA occurence (Jauhar, 1993b; Catalán et al., 2004; Cao et al., 2008). Of this complex we analyzed the species *L. multiflorum* (cultivar Lema), *L. temulentum*, *L. rigidum*, *L. remotum*, *F. pratensis*, and *F. rubra*. Individuals of *L. perenne* (in this study: cultivar Fennema) and *F. arundinacea*, for which PA biosynthesis was described (Koulman et al., 2008) served as positive controls.

To quantify thesinine-conjugates for a comparison of PA content between samples, we used the PA retrorsine as an internal standard (*m/z* 352, **Figure 1A**). To confirm stability of LC-MS measurements, we repeatedly measured so-called quality control (QC) samples (a mixture of all plant extracts in equal proportions) for minimal variation in their chromatograms (Xu et al., 2007; European Medicines Agency, 2011; Demetrowitsch et al., 2015). Comparison of peak intensities of five randomly chosen *m/z* signals in addition to our compounds of interest revealed only little variation between QC measurements with relative standard deviations of peak intensities lower than 15%, except for *m/z* 437.2 (603 s). The value of 15% has been suggested as the upper limit for standard deviation by EMA (Supplementary Table S1; European Medicines Agency, 2011). In addition, we performed a principal component analysis (PCA) on the intensities of *m/z*-t_R_ pairs (the so-called buckets) to further confirm measurement stability. The PCA plot revealed an expected central aggregation of QC samples, and otherwise showed no significant batch bias or other technical artifacts (Supplementary Figure S2).

**FIGURE 2:**
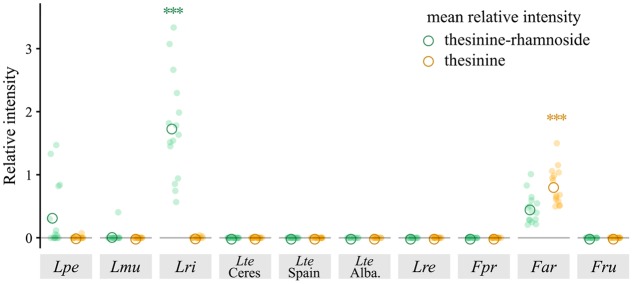
Thesinine-rhamnoside and thesinine in various *Lolium* and *Festuca* species. Relative intensity represents the peak intensities as determined in LC-MS analyses for thesinine-rhamnoside (434.2 m/z, 324 s) and thesinine (288.1 m/z, 340 s) relative to the internal standard (retrorsine, 0.375 μg/ml). ANOVA of thesinine-rhamnoside, *p* < 0.001, Tukey *post hoc* tests for *Lri*, adjusted ^∗∗∗^*p* < 0.001, ANOVA of thesinine, ^∗∗∗^*p* < 0.001 Tukey *post hoc* tests for *Far*, adjusted ^∗∗∗^*p* < 0.001. *Lpe* – *L. perenne* (Fennema), *Lmu* – *L. multiflorum* (Lema), *Lri* – *L. rigidum*, *Lte* – *L. temulentum*, *Lre* – *L. remotum*, *Fpr* – *F. pratensis*, *Far* – *F. arundinacea*, *Fru* – *F. rubra*.

For the following quantitative analyses we calculated peak intensities of thesinine-rhamnoside (we focused on the stereoisomer eluting at 324 s) and thesinine relative to the internal standard (referred to as the relative intensity in the present study). Comparison of the average relative intensities calculated for each species showed widely varying levels of thesinine-rhamnoside and thesinine between the different species (**Figure 2**). *L. rigidum* showed by far the highest average relative intensities for thesinine-rhamnoside in comparison with the other tested species (adjusted *p* < 0.001, ANOVA, Tukey’s test). The prominent presence of thesinine-conjugates in this species and in *L. multiflorum* is a novel observation. *L. perenne* and *F. arundinacea* displayed intermediate levels of thesinine-rhamnoside, while *L. multiflorum* on average contained the least amount of the alkaloid per biomass (**Figure 2**).

The aglycone thesinine was only found in significant amounts in *F. arundinacea* (adjusted *p* < 0.001, ANOVA, Tukey’s test); lower intensities (up to 2500 counts) were recorded in some samples of the other species. As thesinine was only detected in those *Lolium* samples that also contained thesinine-rhamnoside, it is probably the result of thesinine-conjugate hydrolysis taking place in the extract.

**FIGURE 3:**
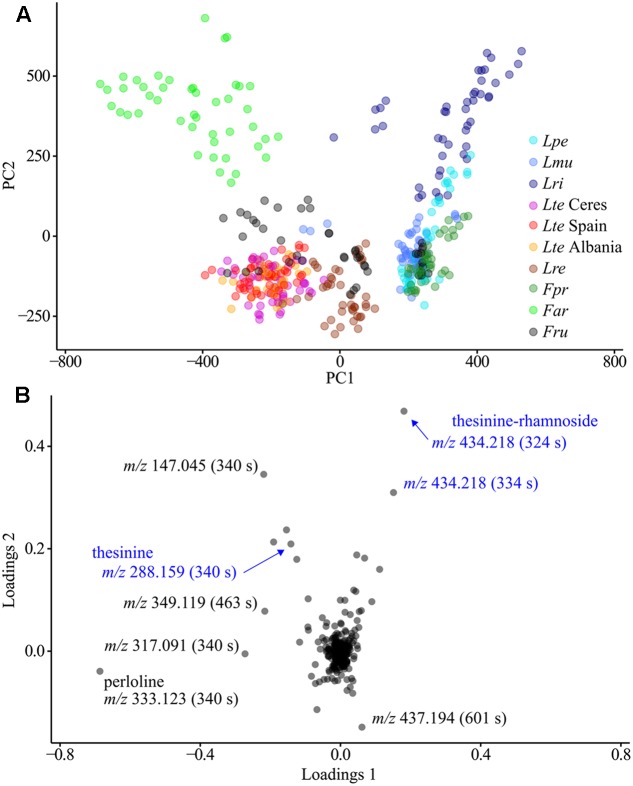
Principal component analysis of all plant extract LC-MS analyses. **(A)** Scores plot, coloring according to species. **(B)** Loadings plot with notable compounds marked. QC samples measurements, outliers and buckets representing internal standards are excluded. *Lpe* – *L. perenne* (Fennema), *Lmu* – *L. multiflorum* (Lema), *Lri* – *L. rigidum*, *Lte* – *L. temulentum*, *Lre* – *L. remotum*, *Fpr* – *F. pratensis*, *Far* – *F. arundinacea*, *Fru* – *F. rubra*.

Another PCA performed exclusively on biological MS data (i.e., without QC sample measurements and excluding internal standards) revealed a strong clustering of samples by species (**Figure 3**). The corresponding loadings revealed that the difference between species was attributable to varying levels of perloline (*m/z* 333.123, 340 s), a diazaphenanthrene alkaloid known from *L. perenne* and *F. arundinacea* metabolomes (Bush and Jeffreys, 1975; Cao et al., 2008). In addition, the thesinine-conjugates are among the 10 buckets with the highest loadings meaning that their variability also greatly contributes to the species-specific differences in metabolic composition.

### Variable Occurrence Patterns of Thesinine Conjugates between Species

**FIGURE 4:**
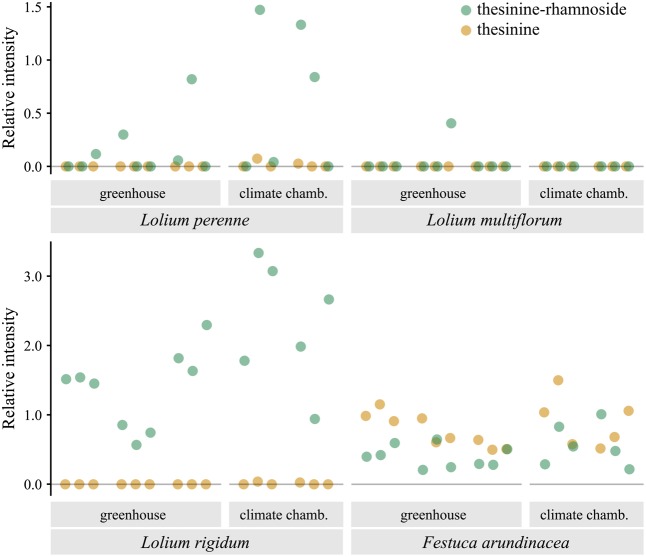
Occurrence of thesinine-rhamnoside and thesinine in individual samples of *L. perenne*, *L. multiflorum*, *L. rigidum*, and *F. arundinacea*. Intensities extracted from LC-MS data for thesinine-rhamnoside (*m/z* 434.2, 324 s, green) and thesinine (*m/z* 288.1, 340 s, orange) displayed relative to the intensity of an internal standard (retrorsine, 0.375 μg/ml). Samples are grouped according the place in which the grass plants were grown and the growth stage at sampling (2, 6, and 8 weeks after germination).

Analysis of samples taken from various individuals of the PA containing species for their thesinine-conjugate content revealed an unexpected pattern of intra-species variation with respect to the thesinine-rhamnosides (**Figure 4**). Whereas all sampled *L. rigidum* and *F. arundinacea* individuals contained levels of the alkaloid above the detection limit, the occurrence of thesinine-rhamnoside is variable in individuals of *L. perenne* and *L. multiflorum*. The alkaloid was only detected in eight out of 15 tested *L. perenne* plants and, even more remarkably, only in one out of 15 *L. multiflorum* plants, which thereby explained the low average relative intensity observed for that species (**Figures 2**, **4**).

Pyrrolizidine alkaloid biosynthesis is known to be coupled to plant growth in various species and is localized in specific tissues and cells, resulting in an individual spatio-temporal pattern of PA accumulation (Anke et al., 2004, 2008). By analyzing plants at various growth stages (2, 6, and 8 weeks after germination), we detected increasing amounts of total PAs, suggesting a constitutively active PA biosynthesis and accumulation in *L. rigidum* and *F. arundinacea*. Levels of thesinine-rhamnoside (with a t_R_ of 324 s) and thesinine relative to the biomass did not vary significantly at the diverse growth stages (ANOVA, *p* > 0.05) indicating that, on average, PA levels per biomass appear constant throughout plant development. Whereas for *L. perenne*, thesinine-rhamnoside occurrence is sporadic, we detected no growth stage, for which not at least one plant individual contained thesinine-rhamnoside, showing that this species is capable of PA biosynthesis as early as the three-leaf stage of the grass (2 weeks after germination).

### The Occurrence of Thesinine-Rhamnoside Is Not Restricted to Specific Cultivars of *L. perenne* and *L. multiflorum*

The striking sporadic occurrence of the PAs in *L. perenne* and *L. multiflorum* motivated us to test whether this pattern is a specific property of the tested cultivars. We therefore expanded the LC-MS analyses to include a variety of cultivars of both species. For *L. perenne*, we choose the cultivar ‘Chicago’ in addition to ‘Fennema’ (see section “Approach for the Detection of Thesinine and Its Conjugates via LC-QTOF-MS”), whereas for *L. multiflorum*, we screened the cultivars ‘Lema’ (see section “Approach for the Detection of Thesinine and Its Conjugates via LC-QTOF-MS”), ‘Pilgrim,’ ‘Lipo,’ and ‘Fabio.’ All cultivars were grown in parallel in the greenhouse and sampled 7 weeks after germination. We used the same LC-QTOF-MS approach as described above, including the use of QCs to show measurement stability (Supplementary Table S1).

**FIGURE 5:**
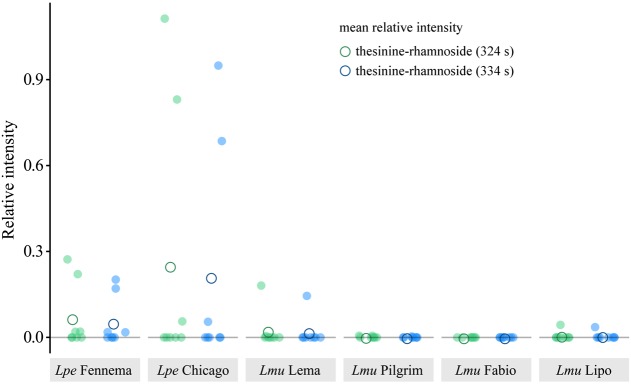
Relative intensity of thesinine-rhamnosides (1 and 2) in various *L. perenne* and *L. multiflorum* cultivars. Relative intensity is the peak intensity as determined in LC-MS analyses for thesinine-rhamnoside stereoisomers (*m/z* 434.2, 324 and 334 s, blue and green, respectively) normalized over an internal standard (retrorsine, 0.32 μg/ml). *Lpe* – *L. perenne, Lmu* – *L. multiflorum.*

Both *L. perenne* cultivars showed, on average, higher relative intensities for the thesinine-rhamnosides than the *L. multiflorum* cultivars (**Figure 5**). Quantification of thesinine-rhamnoside levels in the samples of plant individuals confirmed the sporadic occurrence of the alkaloid in the two species: about half of the analyzed individuals of *L. perenne* contained thesinine-rhamnosides (four plants out of eight for ‘Fennema’ and three out of eight for ‘Chicago’). For the various *L. multiflorum* cultivars, thesinine-rhamnosides were only detected a total of five times out of the 32 individuals (2 for ‘Lema,’ 2 for ‘Pligrim,’ 0 for ‘Fabio,’ and 1 for ‘Lipo’).

### PA Occurrence within Grasses Is Limited to Thesinine-Conjugates and to a Few Pooideae Species

In addition to the analyses focusing on the thesinine-conjugates we also screened the Pooideae species for potential accumulation of other PAs. These analyses included not only the *Lolium* and *Festuca* species (and cultivars) that we have described so far, but also the species *D. glomerata*, *H. lanatus* and *T. aestivum*. *Brachypodium distachyon* was included as a species that split off from other Pooideae before the evolution of the Pooideae-specific *hss* gene (Wesseling et al., unpublished data) and serves as a negative control in this experiment. To enrich potentially occurring PAs and to increase their chance of detection, plant extracts were applied to strong cation exchange solid-phase extraction (SCX-SPE) columns before being subjected to LC-QTOF-MS analysis. The SCX-SPE method was shown to be suitable for the extraction of PAs in their different oxidation states, i.e., tertiary PAs and their respective *N*-oxides (Mroczek et al., 2002; Betteridge et al., 2005; Colegate et al., 2005). We found that the method was also applicable for the extraction of glycosylated thesinines making it a convenient method for extracting and detecting the complete PA repertoire of a plant. To identify PAs, the fragmentation mass spectra of prominent peaks were inspected for typical necine base fragments. Whereas several prominent peaks were apparent in chromatograms of the various plant extracts, no fragments typical for necine bases of PAs were found (except that of the thesinine-conjugates) suggesting that the PA spectrum of grasses tested in this study is limited to the thesinine-conjugates (Supplementary Table S3).

## Discussion

Despite the agronomical importance of grasses, little is known about PAs that are not the product of endophytic fungi. To shed light on this aspect of PA biosynthesis, we have analyzed the PA occurrence in selected species of cool-season grasses (Pooideae, Poaceae). We performed LC-MS-based screening experiments to test a variety of species from this taxon and found only few incidences of PA production (**Figure 2**). In our surveys, PA accumulation was limited to four species placed in the so-called broad-leaved fescue lineage of the *Lolium-Festuca* species complex. The three PA-producing *Lolium* species, namely *L. perenne*, *L. multiflorum*, and *L. rigidum*, are outbreeders and have been shown to be closely related to each other (Catalán et al., 2004; Cheng et al., 2015). These outbreeding *Lolium* species present a sister group to the inbreeding *Lolium* species which include *L. temulentum* and *L. remotum* and which we have shown here to be PA-free. The PA-producing species *F. arundinacea*, however, presents an exception to this pattern. It is also a broad-leaved fescue but of the subgenus *Schedonorus*, which forms a sister taxon to *Lolium* (Catalán et al., 2004; Cheng et al., 2015). The presence of PAs in *F. arundinacea* is surprising considering their absence in *F. pratensis*. The two species have been shown to be closely related to each other, as one of the *F. arundinacea* subgenomes is derived from an *F. pratensis* ancestor (Humphreys et al., 1995). This unexpected pattern of PA occurrence can be explained either by the independent evolution of PA biosynthesis in *F. arundinacea* or by the secondary loss of it in the inbreeding *Lolium* species. Another possibility is that the plant acquired the ability to produce PAs through introgression from a PA-producing *Lolium* species. A comprehensive record of natural and synthetic hybridizations exists between *Festuca* species of the subgenus *Schedonorus*, to which *F. arundinacea* belongs, and the outbreeding *Lolium* species (Jauhar, 1993a). Indeed, several instances of introgression have already been documented (Humphreys and Thomas, 1993; King et al., 1998; Humphreys et al., 2003; Kopecký et al., 2008). Therefore, the genetic components required for PA biosynthesis might conceivably have reached the *F. arundinacea* genome through this mechanism. Detailed genetic and genomic analyses will be needed to clarify this.

Nevertheless, our observation, that PAs are found in one *Festuca* and several *Lolium* species, which include the crop weed *L. rigidum*, suggests that the ability to produce PAs appeared before the evolution of the extant outbreeding *Lolium* species and is thus not a result of selective plant breeding processes as has previously been suggested (Koulman et al., 2008). This is also supported by the *dhs/hss* phylogeny which places the origin of the *hss* gene early during Pooideae evolution (Wesseling and Ober, unpublished data). In contrast to the limited occurrence of PA within the Pooideae, functional *hss* genes are widespread in the genomes of this taxon. The *hss* gene has been identified, amongst others, for *D. glomerata*, *H. lanatus*, and *T. aestivum* in addition to *L. temulentum* and *F. rubra* (Wesseling and Ober, unpublished data), which all tested negative for PAs in this study.

Remarkably, the PA spectrum that we have detected in the various grass species and cultivars is limited to thesinine and its conjugates. PA-producing species of the dicots are often characterized by bouquets of structurally related PAs (Hartmann and Witte, 1995). Despite the structural diversity of known PAs with around 350 different structures having been described in the literature (not including the respective *N*-oxides) (Roeder, 2000), the occurrence of glycosylated PAs is rather unusual. Apart from grasses, they have also been extracted from *Borago* seeds. Of note, in *Borago* the aglycone of these PAs is also thesinine (Herrmann et al., 2002).

Despite the limited number of PA-producing grass species, we have observed an unexpected inter- and intra-specific variability in PA levels and occurrences. First, the analyzed PA intensities are highly variable (**Figure 2**), and the co-occurrence of thesinine and thesinine-rhamnosides are not identical (**Figure 4**). Within *F. arundinacea*, for example, the aglycone thesinine is prominently present, in addition to the thesinine-rhamnosides. Second, PAs were only sporadically found in individuals of *L. perenne* and *L. multiflorum*, whereas each tested individual of *L. rigidum* and *F. arundinacea* contained detectable amounts of PAs. As the plants tested in this study were only a few weeks old, we cannot exclude that PA accumulation is more common in later developmental stages. Even so, our data demonstrate the variability between species in that PA production seems to be a constitutive trait in some species, whereas in others, it occurs only sporadically and might possibly be induced.

In conclusion, our data show that at least four species of the *Lolium-Festuca* species complex are able to produce thesinine conjugates and that there are no hints for further PA structures produced by other species of the Pooidea subfamily. Unexpectedly, the occurrence of thesinine conjugates is sporadic in individuals of *L. perenne* and *L. multiflorum* cultivars, raising questions concerning the evolutionary history of PA production and also about the function that these alkaloids serve in representatives of this taxon. Further studies are required to shed more light onto PA biosynthesis in grasses.

## Materials and Methods

### General Experimental Procedures

Plant extract samples were analyzed by an LC-coupled QTOF-MS set-up. Analytes were separated on a reversed-phase LC column (Nucleodur C18 Gravity column, 100 mm × 2 mm, 1.8 μm, Macherey-Nagel) installed in an Infinity 1260 UHPLC system (Agilent Technologies). A solvent gradient was used at a flow rate of 250 μl min^-1^ (solvent A: H_2_O, 0.1% HCOOH, solvent B: CH_3_CN, 0.1% HCOOH). The LC gradient began at 0% solvent B starting at 1 min and was ramped to 90% over the next 8 min. Column washing at 90% was maintained for 0.5 min and then ramped down to 0% again over 4.5 min the gradient remained for the next 5 min (total runtime of 15 min).

The measurements were performed with the microTOF-QII mass spectrometer (Bruker Daltonik, Bremen, Germany) equipped with an electrospray ionization (ESI) source. The ionization was conducted in the positive ionization mode with a broadband collision-induced dissociation method (bbCID). The source parameters were: dry gas at a temperature of 210°C with a flow rate of 6 l/min and a nebulizer pressure of 1 bar. LC-MS data were further processed by using the Find Molecular Features (FMF) algorithm implemented in Data Analysis 4.2 (Bruker Daltonics) by using the following peak finding parameters: *S/N* = 3, correlation coefficient threshold = 0.7, minimum compound length = 7 spectra, smoothing width = 1.

### Plant Material

For our screening experiments we used a selection of Pooideae species. All species used in this study are summarized in Supplementary Table S2 including information on cultivars and origin. Seeds were placed on wet filter paper until germination. When 2–3 days old, seedlings were transferred to soil-filled pots (Einheitserde Classic, Einheitserde Werkverband e.V.) and then kept either in a greenhouse or climate chamber. For the surveys, the above-ground parts of the grasses were sampled as a whole, flash-frozen in liquid nitrogen, and stored at -20°C.

### Thesinine-Conjugate Screening in *Lolium*-*Festuca* Species Complex

To screen for thesinine-conjugates in members of the *Lolium-Festuca* species complex we grew *L. perenne* ‘Fennema,’ *L. multiflorum* ‘Lema,’ *L. remotum*, *L. rigidum*, and three *L. temulentum* varieties (‘Ceres,’ ‘Albania,’ and ‘Spain’) plus *F. arundinacea* ‘Arola,’ *F. pratensis*, and *F. rubra* in two lots: in a greenhouse (September/October 2014 in Kiel, Germany) and a climate chamber (16 h light and 21°C, 8 h dark and 18°C). Exemplary photos of selected plant individuals are shown in Supplementary Table S4. After 2, 6, and 8 weeks, three plants from each lot and of every species/cultivar were sampled as biological replicates. Flash-frozen plant material was freeze-dried and milled to produce a fine powder, of which 10.0 mg was then extracted with 2.0 ml of 75% MeOH + 0.1% FA.

To be able to assess LC-MS measurement stability we made use of QC samples following the protocol of Demetrowitsch et al. (2015). For this, 20 μl of each plant extract was pooled to create a QC sample, which was then always subjected to the same preparation procedure and freeze-thaw cycles samples as the plant extract samples. Samples were diluted 1:4 (25 + 75 μl) with MeOH spiked with a pyrrolizidine alkaloid as an internal standard (retrorsine, Sigma–Aldrich) at a final concentration of 0.375 μg/ml. All samples were stored at -80°C until the day of measurement. For LC-MS analyses, the samples were divided into three batches, each containing one of the biological replicates, and each being measured three times (technical replicates) within 43 h of thawing. Before each batch measurement, the QTOF was cleaned following the protocol of Demetrowitsch et al. (2015), and at the beginning of each measurement cycle, the QC sample was injected twice for column equilibration (measurements were not included in subsequent data analyses). The QC samples were injected again after every 20th measurement followed by a blank sample (MeOH).

All samples were recorded in the bbCID (broadband collision-induced dissociation) mode providing both MS and MSn spectra at a detection rate of 4 Hz and a collision energy of 10 eV for MS and 20 eV for MSn results.

After data acquisition, we compared internal standard intensities of all runs and excluded obvious outlier measurements from further analyses. Next, the intensity of nine marker compounds in the QC measurements was compared, and their relative standard deviation was calculated to assess overall measurement stability: *m/z* 434.2, 324 s, *m/z* 434.2, 334 s, *m/z* 288.1, 341 s, *m/z* 352.2, 292 s, *m/z* 381.1, 56 s, *m/z* 319.1, 314 s, *m/z* 163.0, 317 s, *m/z* 303.0, 364 s, and *m/z* 349.1, 463 s (see Supplementary Table S1).

For relative quantification of the PAs, their peak intensities were extracted by using the Compass PathwayScreener 1.0 (Bruker Daltonics). The automatization parameters for data evaluation were an extracted ion chromatogram width of ± 5 mDa, with the mSigma tolerance being set to 1000 and the area and intensity threshold being set to 100 counts. The minimum peak valley was set to 1% by a sensitivity level of 99% and a smoothing width of 0.2. We extracted a complete list of intensities for every found peak and manually checked for their accuracy. For relative quantification, we divided the peak intensities with that of the according internal standard. Finally, the mean was calculated from the technical replicates (referred to, in this study, as the relative intensity). ANOVAs and the Bartlett test were performed by using the R stats package version 3.2.3 (R Core Team, 2013). Normal distribution of residuals was checked visually.

Bucket tables were calculated in ProfileAnalysis 2.1 (Bruker Daltonics) by using the following parameters: mass range of *m/z* 100 to *m/z* 750, retention time range 20–720 s, bucket-pair calculation by advanced bucketing with a time range of 5 s and a mass range of 5 mDa. Data were normalized using the quantile algorithm. PCAs were calculated in R by using the functions ‘prep’ for Pareto scaling (pcaMethods package, Stacklies et al., 2007) and ‘prcomp’ principal component calculation (R stats package, R Core Team, 2013). The R stats package was also used to calculate MANOVAs.

### Thesinine-Conjugate Screening in *L. perenne* and *L. multiflorum* Cultivars

Plants of two *L. perenne* and four L. *multiflorum* cultivars were grown in the greenhouse (June/July 2015) and sampled 7 weeks after germination. Sample preparation, data acquisition, and data analysis largely followed the same protocol as described above. Plant extracts were diluted 1:5 (50 + 200 μl), and the final concentration of the internal standard retrorsine was 0.32 μg/ml. All samples and QC samples were analyzed via LC-MS in three batches that were each measured within 24 h of thawing. For the assessment of measurement stability the following signals were chosen: *m/z* 381.1, 57 s, *m/z* 119.1, 124 s, *m/z* 163.0, 319 s, *m/z* 333.1, 340 s, and *m/z* 437.2, 603 s in addition to *m/z* 434.2, 324 s, *m/z* 434.2, 334 s, and the internal standard *m/z* 352.2, 292 s.

### Screening for Other Pyrrolizidine Alkaloids in Various Pooideae Species

The Pooideae species selected included all the *Lolium* and *Festuca* species and cultivars plus *D. glomerata*, *H. lanatus*, *T. aestivum*, and *B. distachyon* (see section “Plant Material” and Supplementary Table S2). Powder from various plant parts and growth stages of a specific species were mixed in approximately same proportions and were extracted and prepared based on the protocol of Betteridge et al. (2005). In short, plant material stored at -20°C (fresh and lyophilized) was powdered before extraction with 0.1 N H_2_SO_4_ (10 ml per 0.1 g freeze-dried material and approx. 2 ml per 0.1 g fresh material). Heliotrine (0.8 μg, Latoxan) and retrorsine (0.8 μg, Sigma–Aldrich) were added as controls to the samples. The extracts were applied to SCX-SPE columns (Phenomenex) that were treated according to the manufacturer’s instructions. Alkaloids were eluted with ammoniated MeOH, which was subsequently evaporated to dryness. The residue was resolved in 2.0 ml MeOH and again diluted 1:20 (10 + 190 μl) and analyzed via LC-MS (AutoMSn mode). The LC-MS chromatograms were manually screened for potential necine base fragments: *m/z* 156, *m/z* 138, *m/z* 120 for retronecine (and isomers), *m/z* 168, *m/z* 150 for otonecine, and *m/z* 142, *m/z* 124 for isoretronecanol (and isomers) (Colegate et al., 2005; Zhou et al., 2010).

## Author Contributions

The study presented here was designed by A-MW and DO. A-MW cultivated the plants and prepared plant extracts. LC-MS measurements were optimized, planned and conducted by A-MW and TD. The data were analyzed by A-MW who also wrote the manuscript with assistance from TD, KS and DO. The authors would also like to acknowledge the help of a professional editing service.

## Conflict of Interest Statement

The authors declare that the research was conducted in the absence of any commercial or financial relationships that could be construed as a potential conflict of interest.
