# Supplementary material for: Variability of Pyrrolizidine Alkaloid Occurrence in Species of the Grass Subfamily Pooideae (Poaceae)
Source: Front Plant Sci. 2017 Nov 30;8:2046. doi: 10.3389/fpls.2017.02046 (PMC5714882; doi:10.3389/fpls.2017.02046)
Supplement: Supplementary file 1 [file Data_Sheet_1.PDF]

Supplementary material

Variability of Pyrrolizidine Alkaloid Occurrence in Species of the Grass Subfamily Pooideae (Poaceae)

Anne-Maria Wesseling[1], Tobias J. Demetrowitsch[2], Karin Schwarz[2], Dietrich Ober[1]*

***Correspondence:** Dietrich Ober: dober@bot.uni-kiel.de

1 Supplementary Figures and Tables

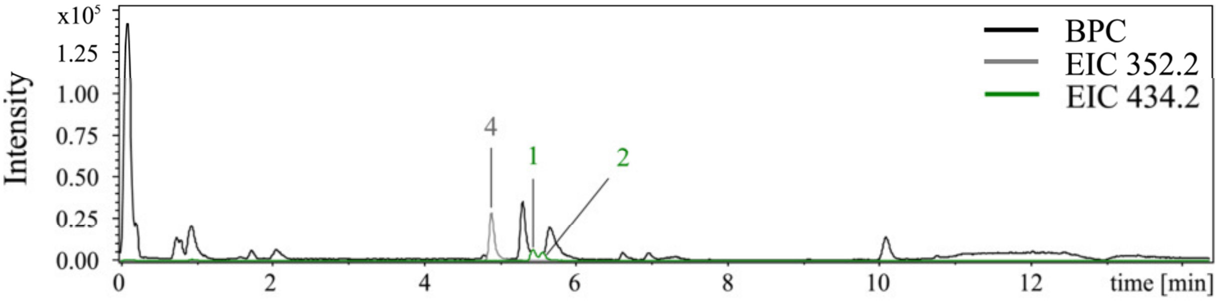

Supplementary Figure S1: Base peak chromatogram (BPC) of a *Lolium perenne* extract recorded via LC-QTOF-MS. Extracted ion chromatograms (EICs, smoothed) depicting the elution profiles of the thesinine-rhamnosides (*m/z* 434.218, 1 and 2) and the internal standard retrorsine (*m/z* 352.173, 4).

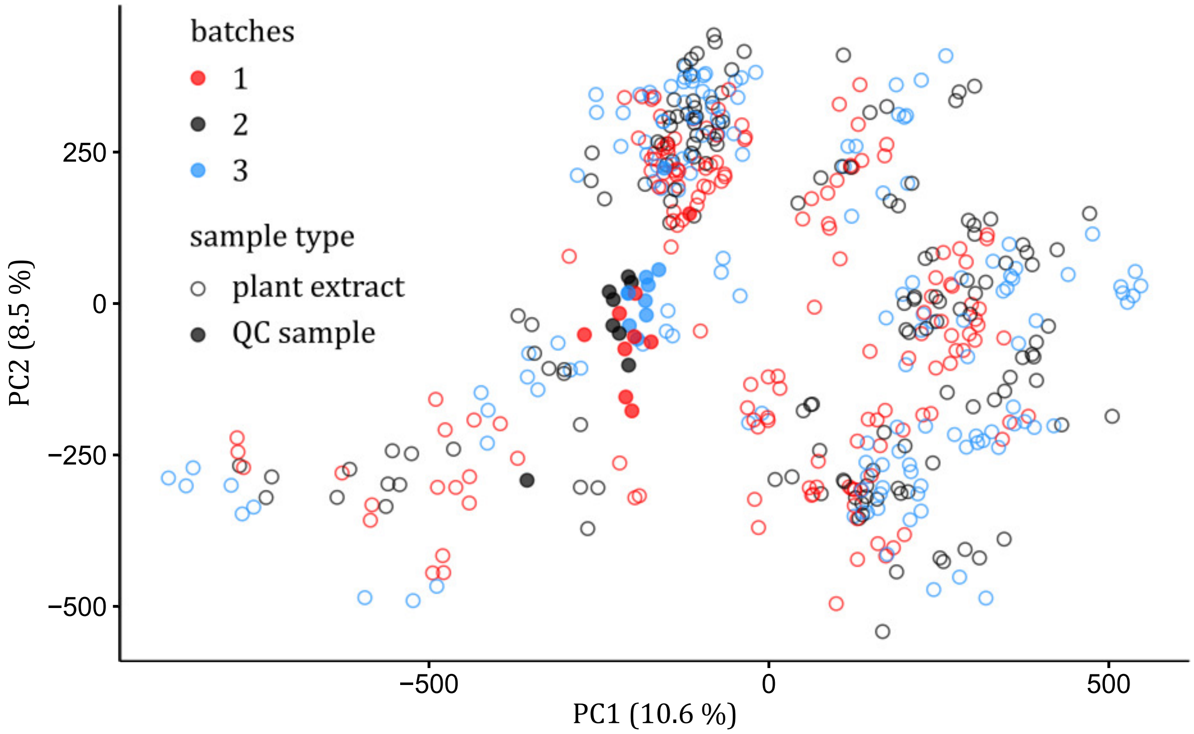

Supplementary Figure S2. Principal component analysis of the complete LC-MS dataset of the *Lolium-Festuca* screening. MANOVA of PC1 and PC2 scores reveals that there are no significant differences between batches ($p = 0.31$)

Supplementary Table S1. Stability of marker compounds in quality control (QC) samples throughout the LC-QTOF-MS analysis for the *Lolium-Festuca* screen

Marker compound	Mean intensity	Relative standard deviation	Maximum RT difference
***Lolium-Festuca* species screen for thesinine and thesinine-rhamnoside**			
**Thesinine-rhamnoside (1)**<br>434.2 m/z, 324 s	10224	7.0%	3.1 s
**Thesinine-rhamnoside (2)**<br>434.2 m/z, 334 s	4699	8.8%	1.6 s
**Thesinine (3)**<br>288.1 m/z, 341 s	3966	7.9%	1.3 s
**Retrorsine (4)**<br>352.2 m/z, 292 s	36256	11.3%	1.8 s
**381.1 m/z, 56 s**	23103	7.2%	1.6 s
**319.1 m/z, 314 s**	7951	13.6%	3.5 s
**163.0 m/z, 317 s**	19540	5.7%	6.6 s
**303.0 m/z, 364 s**	11027	11.2 %	5.6 s
**349.1 m/z, 463 s**	7178	8.2%	3.6 s
***Lolium* cultivar screen for thesinine-rhamnoside**			
**Thesinine-rhamnoside (1)**<br>434.2 m/z, 326 s	1316	12.6%	5.5 s
**Thesinine-rhamnoside (2)**<br>434.2 m/z, 335 s	1117	8.2%	5.1 s
**Retrorsine (4)**<br>352.2 m/z, 294 s	29922	5.9%	5.0 s
**381.1 m/z, 57 s**	17559	5.3%	2.5 s
**119.1 m/z, 124 s**	2578	9.5%	12.1 s
**163.0 m/z, 319 s**	17151	3.1%	6.0 s
**333.1 m/z, 340 s**	28526	6.6%	7.6 s
**437.2 m/z, 603 s**	12466	15.6%	3.2 s

Supplementary Table S2. All Pooideae samples with sources used in this study

Species	Additional information	Source [□]
***Lolium-Festuca* species complex**		
Lolium perenne	Fennema [‡]	NPZ-Lembke
Lolium perenne	Chicago [‡]	NPZ-Lembke
Lolium multiflorum	Lema [‡]	NPZ-Lembke
Lolium multiflorum	Pilgrim [‡]	NPZ-Lembke
Lolium multiflorum	Fabio [‡]	NPZ-Lembke
Lolium multiflorum	Lipo [‡]	NPZ-Lembke
Lolium remotum	GR 11839 [†]	Genebank Gatersleben
Lolium rigidum	GR 11848 [†]	Genebank Gatersleben
Lolium temulentum	Ceres[‡], ABY-Ba 13539-1999U[†]	IBERS Aberystwyth
Lolium temulentum	GR 11902 [†] (Albania [φ])	Genebank Gatersleben
Lolium temulentum	GR 11860 [†] (Spain [φ])	Genebank Gatersleben
Festuca arundinacea	GR 11566 [†], Arola [‡]	Genebank Gatersleben
Festuca pratensis	GR 9987 [†]	Genebank Gatersleben
Festuca rubra ssp. rubra	GR 2197 [†], Engina [‡]	Genebank Gatersleben
**Additional Pooideae species**		
Brachypodium distachyon		Botanical Garden Kiel
Dactylis glomerata		Botanical Garden Kiel
Holcus lanatus	GR 4224[†]	Genebank Gatersleben
Holcus mollis	GR 7269[†]	Genebank Gatersleben
Triticum aestivum var. lutescens	Florida[‡], TRI 29469[†]	Genebank Gatersleben
Triticum aestivum var. lutinflatum	Chinese Spring[‡], TRI 12922[†]	Genebank Gatersleben

[□] full names: Genebank Gatersleben - Genebank Gatersleben of the Leibniz Institute of Plant Genetics and Crop Plant Research (IPK), Germany; NPZ-Lembke - Norddeutsche Pflanzenzucht Hans-Georg Lembke KG, Holtsee, Germany; IBERS Aberystwyth - Genebank IBERS, Aberystwyth University, Wales; Botanical Garden Kiel - Botanischer Garten der Christian-Albrechts-Universität zu Kiel, Germany
[†] accession number; [‡] cultivar; [φ] country of origin

Supplementary Table S3. Summary of results from the general screen for PAs in various Pooideae species. Shown is the presence (+) or absence (−) of LC-MS fragments which could indicate the presence of the most common necine bases: retronecine, otonecine, isoretronecanol or their isomers.

	retronecine			otonecine		isoretronecanol		
**m/z**	**156**	**138**	**120**	**168**	**150**	**142**	**124**	**conclusion**
Lolium perenne	−	−	−	−	−	+	+	→ thesinine-conjugates
L. multiflorum	−	−	−	−	−	+	+	→ thesinine-conjugates
L. remotum	−	−	−	−	−	−	−	no evidence for the presence of PAs
L. rigidum	−	−	−	−	−	+	+	→ thesinine-conjugates
L. temulentum	−	−	−	−	−	−	−	no evidence for the presence of PAs
Festuca arundinacea	−	−	−	−	−	+	+	→ thesinine-conjugates
F. pratensis	−	−	−	−	−	−	−	no evidence for the presence of PAs
F. rubra	−	−	−	−	−	−	−	no evidence for the presence of PAs
Brachypodium distachyon	−	−	−	−	−	−	−	no evidence for the presence of PAs
Dactylis glomerata	−	−	−	−	−	−	−	no evidence for the presence of PAs
Holcus lanatus	−	−	−	−	−	−	−	no evidence for the presence of PAs
H. mollis	−	−	−	−	−	−	−	no evidence for the presence of PAs
Triticum aestivum	−	−	−	−	−	−	−	no evidence for the presence of PAs

Supplementary Table S4. Exemplary photos *Lolium* and *Festuca* species during cultivation in the greenhouse. Pictures were taken at two different growth stages.

	**2 weeks**	**6 weeks**
Lolium perenne	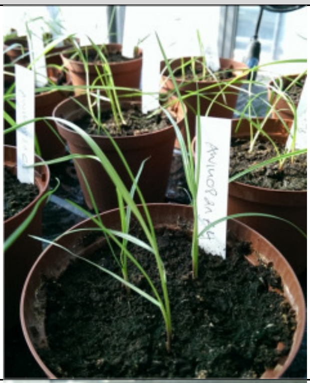	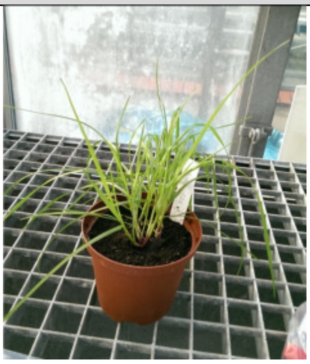
L. multiflorum	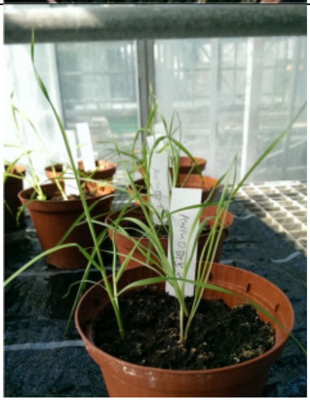	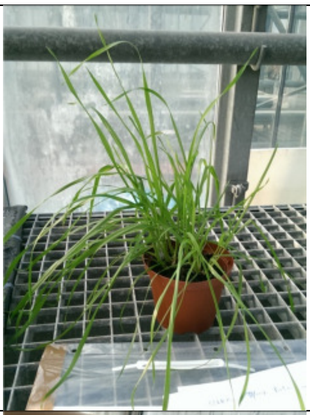
***L. temulentum* (Spain)**	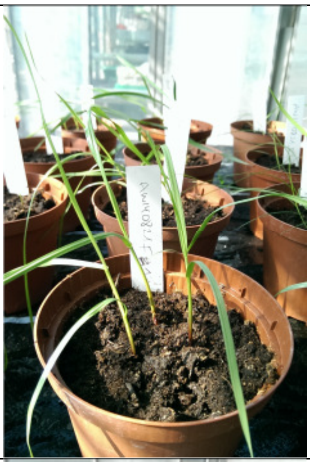	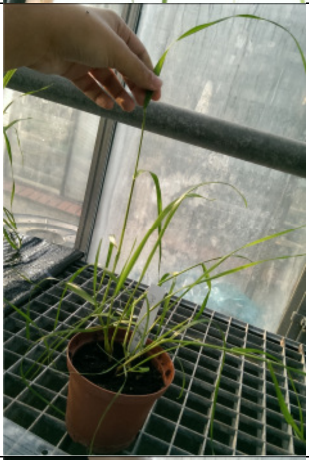
Festuca pratensis	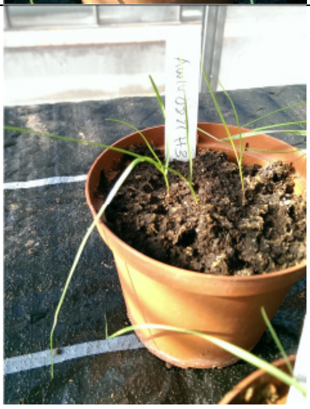	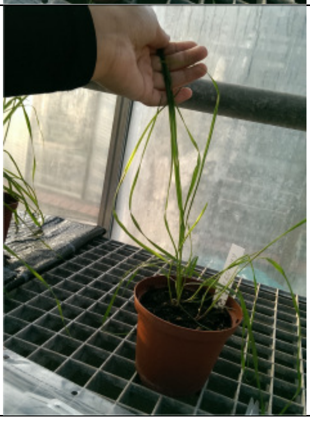
